# Investigating the safety and efficacy of naltrexone for anti-psychotic induced weight gain in severe mental illness: study protocol of a double-blind, randomized, placebo-controlled trial

**DOI:** 10.1186/1471-244X-13-176

**Published:** 2013-06-27

**Authors:** Cenk Tek, Sinan Guloksuz, Vinod H Srihari, Erin L Reutenauer

**Affiliations:** 1 Department of Psychiatry, Yale University School of Medicine, New Haven, CT, USA

**Keywords:** Naltrexone, Schizophrenia, Severe Mental Illness, Obesity, Metabolic Syndrome, Weight Loss, Antipsychotics

## Abstract

**Background:**

Obesity is a growing health problem leading to high rates of mortality and morbidity in patients with severe mental illness (SMI). The increased rate of obesity is largely attributed to antipsychotic use. The effect of antipsychotic medications on H1 and 5HT2 receptors has been associated with weight gain, but there is also a substantial amount of evidence showing that D2 receptor blockade may be responsible for weight gain by interacting with the dopamine-opioid system. Unfortunately, current available medications for weight loss have limited efficacy in this population. Naltrexone, an opioid receptor antagonist, may be a promising agent to reduce antipsychotic induced weight gain by decreasing food cravings. We aim to investigate the safety and efficacy of two doses of naltrexone (25 mg & 50 mg) versus placebo for weight and health risk reduction in overweight and obese individuals (BMI ≥ 28) with SMI, who gained weight while being treated with antipsychotics.

**Methods and design:**

One hundred and forty four patients will be recruited throughout the greater New Haven area. The participants will be randomized to naltrexone 25 mg/day, naltrexone 50 mg/day, or placebo in a 1:1:1 ratio. Participants will be on the study medication for 52 weeks, and assessed weekly for the first 4 weeks and bi-weekly thereafter. The primary outcome measurements are weight reduction and percentage achieving clinically significant weight loss (5% of total body weight). Waist circumference, body mass index, serum lipid profile, fasting glucose, and glycosylated hemoglobin are the secondary outcome measures. The effect of naltrexone on other outcome measurements such as schizophrenia symptoms, depression, dietary consumption, quality of life, cognitive functioning, physical activity, metabolism/inflammation markers, serum leptin, ghrelin, peptide YY, adinopectin, high sensitivity CRP, interleukin 6, interleukin-1B, interleukin-18, and tumor necrosis factor alpha (TNF-α) will be evaluated. The data will be analyzed by applying linear mixed effect models.

**Discussion:**

This is the first large scale study investigating the safety and efficacy of naltrexone in antipsychotic induced weight gain; and hopefully, this may lead to a novel pharmacological option for management of this major health problem.

**Trial registration:**

This trial is registered in http://www.clinicaltrials.gov as NCT01866098

## Background

Obesity is a growing health problem leading to high rates of mortality and morbidity in patients with severe mental illness (SMI) [1-4]. Studies demonstrate the prevalence of obesity and related diseases are much higher in patients with SMI than in general population [2,5-7]. It was found that 42% of individuals with schizophrenia had a Body Mass Index (BMI) of 27 or greater, compared to 27% of the general population [8]. In the Northern Finland 1966 birth cohort study, rates of obesity in patients with schizophrenia were 42% compared to 13% for rest of the cohort [9]. A review of 45 studies including patients with bipolar disorder shows the overall prevalence of overweight and obesity is higher in bipolar patients than in control populations [6]. The increased rates of obesity can be mostly attributed to antipsychotic (AP) medication use. In a landmark study, Allison et al. reviewed available research data and found that, on average, patients gain weight after 10 weeks of treatment with any antipsychotic medication, but that there were differences across medications: patients taking clozapine had a mean weight gain of 3.99 kg, followed by a mean weight gain of 3.51 kg for olanzapine treated patients, 2.10 kg for chlorpromazine, 2.00 kg for risperidone, 0.48 kg for haloperidol, 0.43 kg for fluphenazine, and 0.04 kg for ziprasidone [10]. In the CATIE trial, >7% of baseline weight gain occurred in 30% of patients taking olanzapine over 18 months, but only 7% to 16% of patients taking other antipsychotics had this clinically significant weight gain [11]. Even aripiprazole and ziprasidone, with their more favorable weight gain profiles, produce over 7% total body weight gain in approximately 10% of clinical trial subjects. In a large study of first episode psychosis patients, participants gained an average of 16 kg on olanzapine and 7.5 kg on haloperidol [12]. The mechanism of weight gain associated with the novel antipsychotics has not been fully elucidated, but may be related to blockade of histamine (H1), serotonin 2C (5HT2c) and alpha 1- adrenergic receptors on hypothalamic neurons, resulting in distortion of signals from the gastrointestinal (GI) tract to the brain. [13-16]. While these two receptors may explain the excess weight gain that is observed with newer agents, they fail to explain the weight gain produced by agents like haloperidol, which have no affinity to these receptors. In fact, the sole effect of haloperidol is to block Dopamine 2 (D2) receptors, an action that is shared to a varying degree by all approved antipsychotic agents. Thus, D2 receptor blockade appears to play an additional role in weight gain. D2 receptors are not directly involved with energy regulation; however, they do play an important role in the brain's food reward system. Food is one of the primary substrates of the reward system in higher living organisms. Highly storable energy sources, such as fats and sugars, are extremely enjoyable through the actions of opiate receptors, which are partly mediated by D2 receptors [17]. D2 receptor blockade in animals is shown to produce an effect similar to dilution of the reward itself [18]. Blockade of D2 receptors may make the whole system less sensitive to stimuli, and thereby push the organism to eat more in order to achieve similar levels of enjoyment.

### Reward system, opioid receptors and appetite

Like any non-automatic function that is essential for survival, mammalian feeding behavior is closely modulated by the reward circuits in the brain. Endogenous opioids play an important role in these reward circuits [19]. Following the discovery of endogenous opioids, it was found that opioid receptor antagonists (ORA) decrease self-feeding in food deprived rats [20]. Since then, the animal literature has expanded significantly, and this finding has been replicated in non-food deprived rats and many other mammalian species [20]. It is now fairly clear that ORAs have little effect on initiation of feeding, but rather attenuates preference for “palatable diets” and eating behavior [21]. Injecting animals with opioids increases the preference for high carbohydrate, and even more so, high fat diets, an effect readily blocked by ORAs. There is no evidence that ORAs actually block the taste sensation in animals or humans [20]. Human studies revealed intact taste, but a decrease in the pleasantness of sweets, after administering the ORA naltrexone [19]. Since ORAs block the preference for saccharin solutions in rats, it appears that the macronutrient or caloric content of food does not activate opioid pathways [22]. Other findings show that palatable foods result in endogenous opiate release, related mRNA expression, and opioid receptor occupation in sites of the brain that are critical for both feeding and overall reward circuits, most strikingly in the nucleus accumbens and hypothalamus [19]. The rewarding effects of opioids are exerted mainly through dopamine [17]. Obese human subjects are shown to have significant increases in their circulating β-endorphin levels compared to non-obese controls [23]. Also, more palatable foods are shown to induce higher circulating β-endorphin levels in normal weight human subjects [24]. Increased extracellular dopamine levels occur in the nucleus accumbens and striatum in response to rewarding food stimuli. Notably, obese subjects have been shown to have lower striatal D2 receptor availability at a rate that is inversely proportional to the level of obesity [25-27]. A mechanism for obesity in this population has been proposed here, in which overeating occurs as an attempt to stimulate the dopamine system.

### A working hypothesis of antipsychotic induced weight gain

Patients with schizophrenia treated using antipsychotic medications have been shown to have a preference for diets high in fat and sugar [28]. Patients with schizophrenia also typically seek behaviors that increase dopamine mediated reward in the brain such as smoking and substance use, both of which occur more frequently in this group than in the general population [29]. We propose a model where patients with schizophrenia increase their intake of foods high in sugar and fat in order to increase their low dopaminergic tone, which results from dopamine receptor blockade, via endogenous opioid release. The finding that medicated obese women with schizophrenia have been shown to possess higher circulating β-endorphin levels than matched controls provides partial support for this hypothesis [30]. Medications with additional H1 receptor blockage impair satiety signaling from GI tract to the hypothalamus; thus, there is no “stop” signal for reward eating, which in turn causes further weight gain.

### Clinical trials with the opioid receptor antagonist naltrexone for obesity

Naltrexone is an oral agent that competitively antagonizes all known opioid receptors in the brain. Human studies were completed in individuals with a range of illnesses, including schizophrenia, and naltrexone has been shown to be a safe and easy agent to use. It is shown to decrease craving in alcoholics and is approved by the The Food and Drug Administration (FDA) for the treatment of alcohol dependence [31]. Naltrexone is reported to decrease craving for other substances of abuse, like nicotine. Furthermore, it has been shown to prevent secondary weight gain due to cessation of cigarette smoking at low (25 mg and 50 mg), but not higher doses [32]. Naltrexone has been tested in human feeding studies, and has been shown to reduce both the quantity of food eaten and the choice of palatable foods [19]. A study that was conducted for antidepressant- and lithium-induced weight gain in bipolar women reported weight loss during naltrexone treatment; however, weight on average returned to baseline after cessation of active treatment. Patients reported decrease in food cravings while they received naltrexone, but not while taking placebo in this study [33]. There is also a well-studied bupropion/naltrexone combination for obesity treatment [34,35]. Among several bupropion studies for smoking cessation in schizophrenia concomitant to antipsychotic use, only one –from our center- reported weight effects. In this study, both bupropion and placebo groups gained similar levels of weight regardless of their quit status [36].

### Rationale and significance

In summary, obesity interventions in populations with SMI are urgently needed. Current available medications for this purpose are limited and have not been effective in this population. Antipsychotic medication actions on H1 and 5HT2 receptors only partly explain why they cause weight gain. We developed a working hypothesis that D2 receptor blockade might be partly responsible for weight gain by interacting with the dopamine-opioid system. Thus, we propose a randomized, double blind, placebo controlled study of two different doses of naltrexone (25 mg & 50 mg) for adequate duration to counteract antipsychotic induced weight gain.

### Aims

To determine the efficacy of naltrexone versus placebo for weight and health risk reduction in 144 obese individuals with SMI treated with an antipsychotic medication.

#### Hypothesis 1

Subjects receiving naltrexone 25 mg and 50 mg for 52 weeks will lose weight, and improve BMI and waist circumference, at a greater level than the subjects who receive placebo.

#### Hypothesis 2

Both naltrexone arms will improve health risk markers such as serum lipid profile, fasting glucose, and glycosylated hemoglobin (HbA1c), better than placebo.

#### Exploratory aim

To determine the optimum effective dose of naltrexone to counteract antipsychotic induced weight gain.

## Methods

### Study setting

The study will be run at the Connecticut Mental Health Center (CMHC) and its affiliated satellite clinics throughout the greater New Haven area. The expected duration of the study is 5 years. The study has been approved by The Yale University School of Medicine Human Investigation Committee (HIC#1207010507), and registered at ClinicalTrials.gov (NCT01866098). The study will be conducted in accordance with the Declaration of Helsinki and the International Conference on Harmonization (ICH) - Good Clinical Practice (GCP) guidelines for clinical trials.

### Study population

One hundred and forty four patients with SMI will be recruited from the CMHC and its affiliated satellite clinics throughout the greater New Haven area. All participants will be carefully assessed by physical examinations and laboratory findings. Any medical problem that needs immediate attention will be referred to each participant’s own primary care physician. All potential subjects will meet with study staff for intake and evaluation sessions to determine eligibility prior to enrollment. Eligibility will be determined through participant report, chart review, and communication with their CMHC clinician/psychiatrist, and this will be completed by the Principal Investigator (PI) and research coordinator.

### Recruitment process

Subjects will be recruited through on-site flyers and clinician referrals. The study will also be announced to all the psychiatrists who are attending physicians at the sites, as well as the administrative heads of each clinic through team meetings and email lists. Subjects will be prescreened for eligibility.

### Retention strategy

Based on our experience with this population, and the existing literature, we will implement strategies to minimize attrition. These strategies are: 1) Provider relationship: The clinicians who work in the treatment sites will be thoroughly informed about the study methodology, as well as the study's risks and benefits. The bulk of recruitment will be from the CMHC, where the PI has administrative responsibilities, and has direct access to clinicians, as well as patients. 2) Information: During the consent process, patients will be informed in detail about the risks, benefits, duration, and requirements of the study, as well as the importance of completing follow-ups. Subjects will also be fully informed about the possibility of being assigned to the placebo control group. 3) Flexible scheduling: Assessment appointments will be made around subjects’ schedules, including before or after hours, or right before/after clinical appointments at the same site. Calibrated portable scales will be used for off-site appointments. 4) Transportation: If transportation is not available, bus tokens, bus passes, and city transportation for disabled subjects, when necessary, will be provided. Regular shuttles between sites will be accessible to subjects for appointments. 5) Missed appointments: Participants will receive early reminders both through study staff and from their clinicians. We will discuss missed appointments with the participants, provide alternatives, and express our appreciation of their efforts at every step. 6) Monetary reinforcement: We are going to utilize an incrementally increasing monetary compensation as the participant moves through the study. Every milestone assessment payment will be more than the previous one. Immediate cash payments will be disbursed instead of checks. 7) Lost to follow-up: In case of difficulty contacting subjects, we will utilize clinicians’ help and schedule clinical appointment times based on prior permission from subjects during consent. Assessments in these cases can be done right before or after the clinical appointment instead of scheduling future appointments. If time or other considerations prevent the subject from completing the entire assessment, we will prioritize obtaining subjects’ weight, which is the primary outcome measure, and they will still be partially compensated. 8) Further participant refusal for assessments: After inquiring about the reasons for discontinuing participation in the study, if we cannot solve the immediate problem, we will consider the refusal temporary and situational, and ask for permission to re-contact at a later date. If the patient refuses re-contact, however, we will consider them to have withdrawn from the study. 9) Monitoring Retention: We will implement review of each patient’s participation and risk for loss to follow-up at each team meeting in order to proactively problem solve and implement retention strategies.

### Inclusion/exclusion criteria

#### Inclusion criteria

1). Ages 18 to 75 years old

2). Meet DSM-IV criteria for schizophrenia, schizoaffective disorder, bipolar disorder, major depression, or another psychotic disorder based on SCID interview

3). BMI of 28 or over

4). On a stable dose of antipsychotic medication; i.e. at least one month with no dose change, and three months from an antipsychotic switch

5). Deemed to be symptomatically stable by the clinical staff for the past two months

6). Over 7% total body weight increased on antipsychotics for the subjects within first year of illness

#### Exclusion criteria

1). Meet criteria for current opiate abuse or dependence (confirmed by positive urine drug screen for opiates or, if suspected by study doctor via patient history and or suspicion of occult opiate use, a naloxone challenge will be performed.)

2). Current history of dementia or mental retardation

3). Not capable of giving informed consent for participation in the study

4). Women who are pregnant or breast-feeding

5). Physical conditions affecting body weight (e.g. Cushing’s disease, polycystic ovary syndrome)

6). Diabetes Mellitus (defined as prescribed an anti-diabetic medication for diabetes or a hemoglobin A1c level > 7 confirmed by primary care physician at screening)

7). Severe liver dysfunction (serum aminotransferases greater than three times normal), acute infectious hepatitis, liver failure.

### Study design

After screening for eligibility and signing the informed consent, eligible subjects will be randomized to active Naltrexone 25 mg, Naltrexone 50 mg, or placebo groups in a 1:1:1 ratio using a computer randomization procedure at the CMHC research pharmacy, blind to both research team and subjects. Separate randomization schedules will be applied based on gender and antipsychotic agent to yield an equal number of male and female subjects in each arm. This is to control for possible differential gender effects of Naltrexone on weight as well as differences brought on by higher and lower weight liability medications.

Subjects will take the study medication daily for 52 weeks. Compliance will be monitored by pill counts as well as 25 mg riboflavin added to the capsules which provides florescence to urine under Wood’s lamp (UV or black light) [37,38]. Subjects will be given a study identification card listing the fact that they may be taking naltrexone and this card will include the study emergency cell phone number which will be available 24 hours a day, 7 days per week in case of an emergency. Subjects will be seen weekly for the first 4 weeks of the study; thereafter they will be seen on a bi-weekly (every other week) basis to be assessed (i.e. weight, side effect check, paper questionnaires) throughout the remaining 48 weeks of treatment (Table 1).

**Table 1:** Study flow

MINI, PANSS, Demographics, Physical Exam	Week 0
Urine toxicology, pregnancy test for female subjects, fluorescence check in urine for compliance	Week 0 and every 4 weeks subsequently + as needed determined by study physician
Fasting Glucose, Insulin, HbA1c, Lipid Profile, Liver function tests, FTND	Week 0, 16, 32, 52
Serum leptin, ghrelin, peptite YY, adiponectin, high sensitivity CRP, TNF-α, IL-1B, IL-6, IL-18	Week 0, 16, 52
Framingham Score	Calculated same time with blood draws.
Vitals (Blood Pressure, pulse)	Weekly for first 4 weeks, then every two weeks
Weight, BMI	Weekly for first 4 weeks, then every two weeks
Waist Circumference, 24hr Food Recall, BPRS, BDI, CDSS, C-SSRS, QCSRF, QLS-Q-18, SF-36, RMR, TFEQ, YPAS	Week 0 and every 4 weeks
UKU side effects	Weekly for first 4 weeks, then every two weeks
Cognitive Battery (BACS)	Week 0, Week 16, Week 52

Abbreviations: *BACS* The Brief Assessment of Cognition in Schizophrenia, *BDI* Beck Depression Inventory, *BMI* Body Mass Index, *BPRS* Brief Psychiatric Rating Scale, *CDSS* Calgary Depression Scale for Schizophrenia, *C-SSRS* Calgary Depression Scale for Schizophrenia, *FTND* The Fagerström Test for Nicotine Dependence, *MINI* The Mini-International Neuropsychiatric Interview, *PANSS* Positive and Negative Syndrome Scale, Q*CSRF* The Questionnaire on Craving for Sweet or Rich Foods, *QLS-Q-18* Quality of Life Enjoyment and Satisfaction Questionnaire, *RMR*: Resting Metabolic Rate, *SF-36* SF-36 Health Related Quality of Life Questionnaire, *TFEQ* Three Factor Eating Questionnaire, *YPAS* The Yale Physical Activity Survey.

### Clinical and biochemical assessments

1). Intake interview including demographics, clinical history, and The Mini-International Neuropsychiatric Interview to ascertain diagnosis.

2). Urine toxicology (Utox) screen to rule out substance use and pregnancy tests for female subjects.

3). Weight (Wt) to the nearest 0.1kg and height (ht) will be measured to calculate BMI. Waist circumference (wc) will be measured with a tape measure placed on the midpoint between iliac crest and lowest rib rounded to the nearest 5 mm. Our lab has established procedures for wc measurement (Interrater ICC = 95%). Resting blood pressure (bp) and pulse will be measured.

4). Laboratory testing: All blood sampling is done via an indwelling 21 gauge Intracath catheter placed in an arm or hand vein. Fasting blood samples are obtained from free-flowing blood, taking care to discard fluid from the dead space of the system prior to sampling and to leave the dead space filled with heparinized saline (50 units/ml) after sampling. Blood will be drawn by an institutionally credentialed phlebotomist at the CMHC.

a. Fasting glucose and insulin: Plasma glucose will be analyzed by the glucose oxidase method (Yellow Springs Instruments, Ohio). Plasma immunoreactive insulin concentrations will be determined with a double antibody radioimmunoassay (Diagnostic, Webster, TX).

a. Lipid Profile: Total cholesterol (Total-C), High-density lipoprotein (HDL-C), and triglycerides (TG). Blood will be collected into vacutainers containing no additive. Serum cholesterol (Total-C, HDL-C) and TG will be determined by standard enzymatic procedures (Sigma, St. Louis, MO).

a. Blood will be collected into vacutainers containing EDTA. Assays of HbA1c will be performed using an Ames DCA 2000 analyzer (Miles, Inc., Elkhart, IN).

a. Serum leptin, ghrelin, peptite YY, adiponectin, high sensitivity C-reactive protein (CRP), interleukin 6, interleukin-1B, interleukin-18, tumor necrosis factor alpha levels (TNF-α) will be assayed by Yale Center for Clinical Investigation core laboratories.

5). Brief Psychiatric Rating Scale (BPRS): This is one of the most frequently used overall measure of schizophrenia symptoms. It will be utilized to demonstrate that the naltrexone does not cause any worsening of the illness symptoms apart from the usual fluctuations of the natural course [39].

6). Positive and Negative Syndrome Scale (PANSS): This is a widely used scale to assess the severity of positive and negative symptoms observed in psychosis [40].

7). Beck Depression Inventory (BDI): 21-item version is a widely used measure of the features and symptoms of depression. The BDI taps a broad range of negative affect – not just depression – and is a highly efficient measure for detecting fluctuations in broad psychopathology and distress [41].

8). Three Factor Eating Questionnaire, also known as the Eating Inventory (TFEQ) is a measure of eating behaviors with three factors: dietary (cognitive) restraint, disinhibition, and hunger [42]. The TFEQ is a frequently used measure in obesity trials. The TFEQ has clinical utility to assess changes during treatment as well as treatment outcomes [43]. A 24hr food recall is administered subsequent to TFEQ in our lab protocol.

9). The Questionnaire on Craving for Sweet or Rich Foods (QCSRF): The QCSRF is a two factor, nine-item scale assessing the presence of cravings for rich and sweet foods and has been found to have good psychometric properties [44].

10). The Yale Physical Activity Survey (YPAS): is an interview-administered questionnaire that assesses activity participation in number of hours/week in different categories (i.e. work, recreation, exercise) and takes about 20 minutes to complete [45,46].

11). Resting Metabolic Rate (RMR): In our experience, this group of subjects does not tolerate indirect calorimetry measurements well even with portable devices. Instead, we will utilize the Mifflin-St. Jeor predictive formula, which is shown to be highly predictive of indirect calorimeter measured RMR, and is best among the available formulas for this specific population [47].

12). Quality of Life Enjoyment and Satisfaction Questionnaire (Q-LES-Q-18): The Q-LES-Q-18 is specifically designed to use with schizophrenia patients and focuses on patient’s subjective perception of various aspects of their life [48].

13). SF-36 Health Related Quality of Life Questionnaire (SF-36): a 36-item short-form was constructed to survey health status in the Medical Outcomes Study [49]. The SF-36 was designed for use in clinical practice and research, health policy evaluations, and general population surveys. This instrument is validated for use in patients with schizophrenia [50].

14). UKU Side Effect Checklist (UKU): This is a comprehensive rating scale developed for psychotropic drugs and a cross-sectional study of side effects in neuroleptic-treated patients [51]. Common adverse events related to naltrexone will be added to this scale, as needed.

15). Columbia-Suicide Severity Rating Scale (C-SSRS): This is a scale developed for use in clinical trials of psychoactive agents for the FDA to be used for safety tracking of medications for suicidality [52].

16). Calgary Depression Scale for Schizophrenia (CDSS): This scale is specifically designed for the assessment of depression in schizophrenia and has been used widely [53].

17). The Fagerström Test for Nicotine Dependence (FTND): This is a scale used to assess nicotine dependence [54].

18). Cognitive Battery. The Brief Assessment of Cognition in Schizophrenia (BACS): Given the cognitive impairment of schizophrenia, careful assessment of cognition is necessary for determining the feasibility of using any medication in this population. This is a widely used cognitive battery developed to assess cognition repeatedly in patients with schizophrenia. It was found to be as reliable, sensitive as a standard battery of tests that requires longer time to administer [55,56].

### Statistical analysis

#### Sample size and power calculation

The aim of this study is to compare the efficacy of two different doses of naltrexone to placebo. In our pilot study, an effect size of 1.53 (Cohen’s D) was achieved. The power analysis assumes a similar weight loss difference between naltrexone and the placebo groups, a two-tailed 0.05 significance level, 0.80 power, and the worst case scenario of male subjects not responding to naltrexone at all. A three group power analysis with a significance level of 0.05 (two sided) was conducted using GPower 3.03 software [57] after Cohen’s D was converted to an F value. Then, the sample size was adjusted for post hoc analyses to detect a 1.52 kg difference (1⁄2 SD of naltrexone group in pilot study) between naltrexone 25 mg and 50 mg doses, as well as between the two genders. We will follow our protocol for retaining subjects in the study very closely in order to reduce attrition. However, since this is an unusually long study for this cohort, we will inflate our sample size to adjust for 32% attrition, which was the intervention dropout rate in our similar studies over one year. Thus the total sample size will be 144, with 48 subjects in each arm, which achieves a power of 0.81 if the pilot results hold.

#### Data analysis

Data will be double entered into databases and concordance will be double-checked. Hard copy documents will be examined to verify questionable observations. Missing data will be examined for each variable. Histograms, normal probability plots, and numerical summaries (skewness, kurtosis) will be used to examine distributional assumptions required for the mixed model analysis described below.

Transformations such as the logarithm, square root, and reciprocal will be considered in the event the normality assumption is inadequate. Residual analysis will also be performed to evaluate distributional variance and linearity assumptions. For all analyses, an alpha threshold of 5% (two-sided) will be used to test for statistical significance and will be performed using SAS v9.3 (SAS Institute, Cary, NC).

Analyses will be performed according to the intent-to-treat (ITT) principle where data from any randomized subjects will be analyzed. We will calculate descriptive statistics for participants at baseline, and compare naltrexone to placebo participants using analysis of variance (ANOVA) for normally distributed covariates and chi-square tests for categorical covariates. The impact of baseline variables that demonstrate clinically relevant group differences will be assessed in supportive analyses using covariate adjustment.

#### Approach to missing data

The primary analysis method will use a likelihood-based mixed model, which accommodates incomplete observations and operates under the assumption that the missing data is missing at random (MAR). Missing data patterns, time to withdrawal and reasons for dropout will be compared between two naltrexone and placebo groups. T-tests, cross-tabulations, and logistic regression will be used to evaluate whether withdrawal is dependent on any observed variables. Finally, should missing data patterns and reasons for missing data suggest a bias or confounding, we will conduct sensitivity analyses using pattern mixture models to examine the influence that dropout bias (informative missingness) may have on treatment differences. Multiple imputation (MI) will also be used in the case of data not MAR. Sensitivity analyses will be performed secondarily among study completers.

#### Hypothesis testing

The primary goal of this study is to compare the weight changes resulting from two different doses of naltrexone compared to placebo. The primary ITT analysis will employ a linear mixed effects model (LMM) to determine the effect of treatment status (between-subjects for: naltrexone 25 mg/50 mg vs. placebo) on the trajectory of weight change over time (within-subjects for: study time points). The interaction between treatment and time will be modeled and interpreted using graphical displays and post-hoc contrasts on least square means. The latter will include estimation of group contrasts at each time point and polynomial (linear, etc.) contrasts for time within each group. However, the primary contrasts will be each dose compared with control at 52 weeks. Because the outcome data are correlated (i.e., weight assessed from the same subject at different time points), weight over time will be modeled through the inclusion of random subject effects and/or structured variance-covariance matrices, with the best-fitting structure determined by Bayesian-Schwartz Information criteria (BIC). Supportive analyses will also include covariate adjustment for clinically significant variables (as determined from baseline evaluations and also other treatment variables, such as sessions attended). The above model will be fit using SAS PROC MIXED (Cary, NC), using restricted maximum likelihood (REML). The proportion of subjects achieving 5% weight loss will be compared between the treatment groups with the Fisher’s Exact test. The effect of treatment assignment on the secondary outcomes, such as BMI, waist circumference, serum lipid profile, fasting glucose, and HbA1c will be evaluated using the linear mixed modeling approach described above. Type 1 error for secondary outcomes will be adjusted using the Bonferroni correction, basing the adjustment on the number of conceptually related statistical tests.

#### Other outcomes

Schizophrenia symptoms (BPRS), depression (BDI, CDSS), dietary consumption (TFEQ), exercise (YPAS), quality of life (Q-LES-Q and SF-36), cognition (BACS) and metabolism/inflammation markers will be obtained. Summary statistics (frequencies, percentages, means and standard deviations, and medians and interquartile ranges) will be calculated for naltrexone vs. placebo at each time point. Appropriate parametric (for normally distributed data) and non-parametric (deviation from normality in data distribution) approaches will be used to ascertain clinically meaningful group differences at each time point. Depending upon the distribution of these outcomes, the effect of naltrexone vs. placebo on change over time in each outcome will also be examined with GLMM, making appropriate model selections for either binary or continuous outcomes.

Using UKU Side Effect Checklist and C-SSRS, we will list and summarize side effects and suicidal tendencies reported in each treatment group. Medication changes that could not be avoided during the study will be examined in three groups: no change, change from a higher weight liability to a lower weight liability medication, and vice versa. Weight liability rankings of antipsychotics will be based on earlier reviews [10,58]. Distributions of all variables will be evaluated for normality. Alternative statistical approaches such as transformations or non-parametric tests will be used, as required.

## Discussion

We will be conducting a randomized double blind placebo-controlled study to investigate the safety and efficacy of naltrexone 25 mg/day and naltrexone 50 mg/day for weight and health risk reduction in obese individuals with SMI treated with antipsychotics. To our knowledge, this is the first study investigating the safety and efficacy of naltrexone, a widely available and inexpensive generic oral opioid antagonist, for the treatment of antipsychotic induced weight gain. With this study protocol, we have considered several different important methodological issues. Since FDA obesity intervention guidelines necessitate longer duration studies, we planned the follow-up period to be 52 weeks. The sample size is also calculated in order to reach adequate statistical power. We expect to recruit 144 patients without encountering any problem within the scheduled time period of 5 years, since CMHC and its affiliated satellite clinics provide service to a large population with SMI. A meta-analysis has shown that one-third of subjects drop out of the randomized antipsychotic drug trials [59]. Thus, we expect a similarly high dropout rate for the current study. In order to minimize attrition, we have developed several retention strategies, which are detailed in methods section. Moreover, we will inflate our sample size to adjust for 32% attrition. Therefore, we expect to achieve a sample size providing enough power to determine the efficacy of naltrexone for weight reduction. Lack of compliance in schizophrenia is common: studies demonstrate that around 50% of patients with schizophrenia do not fully comply with the treatment [60], and this may lead to false interpretation of the results. Therefore, compliance will be closely monitored by pill count. Although pill count is one of the most common methods to monitor treatment compliance, it lacks objectivity [61]. Therefore, we aim to monitor treatment compliance by also using riboflavin as a urinary tracer, which is strongly florescent under UV light. Riboflavin at a dosage of 25 mg will be added to the study capsules, and urine will be visually inspected under Wood’s lamp (UV or black light) [37].

Our pilot study demonstrated all patients in the naltrexone arm lost significant weight during the study period; however, in comparison to placebo, it was statistically significant only for patients without diabetes. We cannot conclude the underlying biological mechanism of this finding with our current knowledge, but for this clinical trial, we decided to exclude diabetic patients, based on the findings of our pilot study. The exclusion of patients with diabetes mellitus constitutes a major limitation, particularly making the outcome difficult to generalize to whole population with SMI. However, it should be kept in mind that nearly 80% of the patients with SMI are non-diabetics [62].

Another strength of this study is the evaluation of the effect of naltrexone on several other measurements: schizophrenia symptoms, depression, dietary consumption, quality of life, cognitive functioning, physical activity, metabolism/inflammation markers: serum leptin, ghrelin, peptite YY, adinopectin, high sensitivity CRP, interleukin 6, interleukin-1B, interleukin-18 and tumor necrosis factor alpha. Hopefully, the assessment of different outcome measures may help us to understand the complex, overlapping, and interrelated elements of successful weight loss, and the underlying mechanisms of naltrexone in antipsychotic induced weight gain in SMI.

Overall, we hope that the results of this study may lead to a novel pharmacological option for management of this major health problem that has until now been lacking adequate treatment.

## Competing interests

The authors declare that they have no competing interests.

## Authors’ contributions

All authors made substantial contributions to the conception and design of the trial and were involved in the drafting of the manuscript and/or revising it for important intellectual content. Each author has given final approval of this version to be published.

## Pre-publication history

The pre-publication history for this paper can be accessed here:

http://www.biomedcentral.com/1471-244X/13/176/prepub
